# Motivation for protection in sexual relationship during the COVID-19 quarantine: analysis of the sociodemographic variables of the Iranian population

**DOI:** 10.1186/s12889-022-13475-4

**Published:** 2022-05-26

**Authors:** Raziyeh Maasoumi, Sara Kazemi, Shima Haghani, Fatemeh Zarei

**Affiliations:** 1 Nursing and Midwifery Care Research Center, School of Nursing and Midwifery, Tehran University of Medical Sciences, Tehran, Iran; 2 Department of Reproductive Health, School of Nursing and Midwifery, Tehran University of Medical Sciences, Tehran, Iran; 3 Department of Health Education & Health Promotion, Faculty of Medical Sciences, Tarbiat Modares University, Tehran, Iran; 4 Nursing Care Research Center, Iran University of Medical Sciences, Tehran, Iran; 5 Department of Health Education & Health Promotion, Faculty of Medical Sciences, Tarbiat Modares University, P.O. Box, Tehran, 14115-331 Iran

**Keywords:** Coronavirus, Covid-19, Sexual Relationship, Home Quarantine, Protection Motivation Theory

## Abstract

**Background:**

Coronavirus is an infectious disease that has affected sexual life. Sexual activity has decreased for many people, and couples' physical contact has reduced. The present study aimed to determine the sexual relationship of Iranian people and related factors during the Corona home quarantine by focusing on all constructs of the protective motivation theory.

**Methods:**

This cross-sectional online survey was conducted on Iranian people under in-home quarantine from July to December 2020 during the Covid-19 pandemic. Seven hundred sixteen people were selected by the convenience sampling method. The data was gathered by an ad-hoc tool consisting of demographic information and sexual relationship regarding protection motivation behavior in the Covid-19 pandemic. Participants should be able to complete the online questionnaire because the questionnaires were completed online. One-way ANOVA, independent T, and Pearson correlation tests were used to analyze the data in SPSS 16 software.

**Results:**

Findings indicate the average age of the participants was 37.78 + 8.34 years. Most of them were women (%85.1), married (%91.3) and had a university education (%90.2). In addition, %42.5% of participants had Full-time jobs and %34.2 lived in Tehran. 49.9% obtained information about corona from TV. 63.3% of the participants and their spouses did not catch Covid-19. Employment status was the only variable that was significantly different from sexual relationships regarding protection motivation (*p* = 0.031). Perceived response efficiency with an average of 79.12 had the highest, and perceived self-efficacy with an average of 69.92 had the lowest mean score among the areas of sexual relationship. Among the protection motivation domains of sexual behavior that all had significant correlations, there was no significant correlation between perceived severity and perceived self-efficacy (*p* = 0.067).

**Conclusion and implication:**

Perceived vulnerability is associated with employment status, place of residence, and age. Sexual relationships regarding protection motivations are only associated with employment status. Overall, participants in the present study reported high levels of perceived severity, perceived vulnerability, perceived response efficiency, and perceived costs Therefore, they reported a high level of motivation for protective and preventive behaviors in their sexual relationship. Our findings suggest that future interventions should focus on general cognition by developing appropriate knowledge about the severity and vulnerability of Covid-19 and sexual behaviors, improving perceived response efficiency, and self-efficacy of protective behavior through skills training.

## Introduction

Coronavirus is an infectious disease that has affected all aspects of human life as well as sexual life. Sexual activity has decreased for many people, and couples' physical contact has reduced [1–3]. However, it is reasonable to have sex between partners unless one or both are at risk of infection [4, 5]. There is no reliable data on the coronavirus's stability in the male and female reproductive system, but some case studies on other coronaviruses have shown positive examples of the female reproductive system [6]. Due to the physical proximity of two people during sexual intercourse, the virus can be transmitted through infected droplets from an infected person or a carrier to a healthy person, so in cases of infection or suspected infection, it is recommended not to have sex [7].

Because the global approach to the disease is prevention, quarantine in various ways is the first strategy to combat Covid-19. The Ministry of Health of Iran, which is the main trustee of public health, proposed the home quarantine plan, and with the cooperation of departments and inter-organizations, this plan started from the end of February 2020 and has been emphasized and supported so far [8]. Concerns about getting Covid-19 through sex despite home quarantine is one of the significant concerns of Iranian couples [9]. Therefore, it seems that the study of sexual relations of couples to adopt preventive behaviors in certain situations such as Covid-19 health crisis based on theoretical frameworks of behavior change can be useful. Therefore, recognizing the factors that cause non-compliance with the proposed social controls and challenge the full implementation of public policies based on social isolation is of particular importance.

One of the theories used to study the factors affecting motivation and ultimately the health behavior of individuals is the Protection Motivation Theory. This theory was proposed by Rogers in 1975 based on the value expectation model to explain the effects of fear on health attitudes and behaviors [10]. Here it is assumed that accepting the recommended health behavior is a direct act of motivating oneself to protect oneself. According to protection motivation theory, environmental and personal factors combine to create a potential health threat [11]. This theory consists of two stages of the Threat-Appraisal Process and Coping-Appraisal Process and a two-stage outcome structure called protection motivation or behavior intention [12]. Threat-Appraisal has two constructs of perceived vulnerability and perceived severity, and Coping-Appraisal has three constructs of perceived response efficiency, perceived self-efficacy, and perceived costs. The above five structures can change behavior by changing the structure of protection motivation. According to Rogers, fear stimulates the intention to engage in protective behavior against health hazards through these five structures, which provokes health behavior [11].

According to various studies, the constructs of this theory have predicted different types of preventive and protective behaviors, including smoking cessation behaviors [13], protective behaviors to reduce road accidents [14], prevention of various types of cancer [15], reduction Risk of AIDS [16, 17], the reproductive and sexual health of Iranian adolescents and prevention of high-risk sexual behaviors [18], and prevention of STIs [19, 20].

It seems that public health efforts to combat the high prevalence of human diseases should focus on preventing and changing the awareness, attitude, and intent to change people's behavior at risk, which indicates the role of awareness in performing preventive behaviors. Since the body of current knowledge about Covid-19 is not very strong in all aspects of health, especially couples' sexual relations, and on the other hand, it seems that the constructs of protection motivation theory provide a good basis for studying this issue, the present study focused on all constructs of protective motivation theory and was performed to determine sexual relationship of Iranian people regarding protection motivation and related factors during home quarantine of COVID-19 pandemic. The present study aimed to answer research questions following from two main perspective:

- First, “are there differences between the sociodemographic variables in the constructs of protection motivation in sexual relationships?”.
- Second, “are the protective motivation constructs in sexual relationships related to each other?”.

## Methods

### Design, sample size, and sampling method, participants

This cross-sectional online survey was conducted in Iranian people in home quarantine from July to December 2020 during the Covid-19 pandemic. Sampling was done by convenience method, and all men and women who received the online questionnaire link could participate in the study. Using the Cochran formula (2% sampling error, 0.5 proportion, and a confidence level of 0.95), the minimum sample size was estimated to be 600. Considering 10% of the sample loss probability, the sample size was taken to be 650, and finally, 716 participants completed the questionnaire.

All samples should be Iranian men and women with the experience of a home quarantine period (home quarantine was a plan that the Ministry of Health of Iran started from the beginning of the outbreak of Covid-19 in the country) who could read and write Persian. The sample did not include both members of the couple but they have to be experienced in sexual relationship and have sexual partner. Their marital status could be permanent marriage, temporary marriage, divorce, and widowhood. But all participants had to have a sexual partner during the quarantine period. Participants should be able to complete the online questionnaire because the questionnaires were completed online.

### Instrument

The data collection tool included two sections with sociodemographic variables and the ad-hoc questionnaire about Protection Motivation behavior in COVID-19.

In part one sociodemographic variables consisted of information about COVID-19 sources of information gathering, history of exposure to COVID-19, gender, age, educational status, marital status, living location, job, and spouse' education.

Part two of tool included ad-hoc questionnaire entitled Protection “Protective Motivation Motivative Sexual Relationship Questionnaire in COVID-19” or “PMSRQ-COVID-19”. It was consisted 23 five-point Likert-scale response items (from 'strongly disagree' to 'strongly agree') in 6 dimensions; perceived vulnerability (4-item; e.g.: If my sexual partner or I may contract COVID-19, unprotected sex (not wearing a mask, not observing personal hygiene) can cause illness.), perceived severity(4- item; e.g.: Having sex with my sexual partner affected with COVID-19 is dangerous; Having been infected with or the possibility of being infected with COVID-19 endangers our sexual intimacy.), perceived costs (4- item; e.g.: The fear of contracting COVID-19 during sex leads to a decrease in one’s sexual desire.), perceived response efficacy (4- item; e.g.: I can talk to my sexual partner about the need to abstain from sex for two weeks after experiencing the symptoms of COVID-19 (cough, fever, body aches).), perceived self-efficacy (4- item; e.g.: I'm sure I can maintain sexual excitement in myself and my sexual partner, even without sex, in case I contract or may contract COVID-19.), and protection motivation (behavioral intent) (3- item.: I have decided to avoid any sexual behavior such as kissing to prevent contracting COVID-19; I plan to abstain from sex with my spouse for two weeks if I have symptoms of COVID-19 (such as fever, sore throat, and shortness of breath).

The scale's score ranged from 23 to 115, where the higher the score, the greater the protective motivated sexual behavior in Covid-19. Each domain's scores were standardized based on 0 to 100.The developers reported the content validity ratio (CVR) and content validity index (CVI) of the instrument to be 0.66–0.83 and 0.91–1, respectively. Moreover, Cronbach's alpha reliability and intra-cluster homogeneity of the questionnaire based on the ICC coefficient were calculated 0.64–0.81, and 0.731–0.975, respectively.

### Ethical considerations and implementation

After receiving the code of ethics from the Faculty of Nursing and Midwifery of Tehran University of Medical Sciences (IR.TUMS.REC.2020.545), the target group received an online questionnaire through accessible social networks, e.g., WhatsApp, Telegram, and Instagram. Data collection was carried out with an online questionnaire. The questionnaire could be completed only once by the user. In line with the Helsinki Declaration of Ethical Principles for Medical Research, the participants' confidentiality was preserved. The collected data was not linked to any individual, and personal identifiers were not used during data storage. Therefore, this study's collected data can only be accessed by the research team.

### Data analysis

The collected data were analyzed by SPSS 16 software, and the significance level was considered less than 0.05. Descriptive statistics (frequency, mean and standard deviation) and inferential statistics were used to analyze the data in line with the research objectives. ANOVA and t-tests were also utilized to evaluate the Protection Motivation based on the demographic characteristics. A Pearson correlation test was used to investigate the relationship between Protection Motivation and it’s dimensions. The normality of the variables was tested by the Kolmogorov–Smirnov test.

## Results

The mean age of 716 samples was 37.78 ± 8.34 years. 85.1% were women, 91.3% were married, 42.5% had full-time employment, and 89.8% had a university education. 61.5% were residents the cities other than Tehran, and 49.9% obtained information about Corona from the national media. 69.3% of the subjects and their spouses did not catch Covid-19 (Table 1). Employment status was the only variable that was significantly differenced protective motivated sexual relationship in COVID-19 (*p* = 0.031; Table 2).

**Table 1:** Demographic characteristics of the study subjects (*n* = 716)

Variable		Frequency (%)
**Gender**	Female	609(85.1)
	Male	107(14.9)
**Marital status**	Married	654(91.3)
	Divorced & Widow	38(5.3)
	Temporary marriage	24(3.4)
**Job**	Full-time	304(42.5)
	Part-time	37 (5.2)
	Self-employment	23 (3.2)
	Else	74 (10.3)
	No job	83 (11.6)
	Housewife	195 (27.2)
**Education**	Diploma	70(9.8)
	University	646(90.2)
**Spouse’s Education**	Diploma	146 (21.6)
	University	530 (78.4)
**Live**	Tehran	245 (34.2)
	Another city	440 (61.5)
	Tehran & my spouse other city	17 (2.4)
	Other city & my spouse Tehran	14 (2.0)
**Information**	TV	357 (49.9)
	BBC & VOA	67 (9.4)
	Telegram	143 (20.0)
	Ministry of Health	82 (11.5)
	Else	67 (9.4)
**Corona**	I catch, and my spouse did not	81 (11.3)
	I did not catch it, and my spouse caught	85 (11.9)
	I and my spouse did not catch	496 (69.3)
	I and my spouse caught	54 (7.5)

**Table 2:** Mean and standard deviation of the sexual relationship regarding Protection Motivation according to demographic characteristics (*n* = 716)

Variable		M (SD)	T or F	*p*-value
**Gender**	Female	74.67 (12.91)	0.567	0.571
	Male	73.88 (15.50)		
**Marital status**	Married	74.40 (13.45)	1.81	0.164
	Divorced & Widow	74.02 (12.22)		
	Temporary marriage	79.61 (10.63)		
**Job**	Full-time	75.41 (13.89)	2.468	*0.031
	Part-time	69.12 (9.57)		
	Self-employment	78.26 (12.26)		
	Else	73.67 (10.63)		
	No job	76.24 (13.60)		
	Housewife	73.43 (13.67)		
**Education**	Diploma	74.73 (12.84)	0.118	0.906
	University	74.53 (13.38)		
**Spouse’s Education**	Diploma	74.36 (13.73)	0.282	0.778
	University	74.72 (13.34)		
**Live**	Tehran	73.93 (14.35)	1.805	0.145
	Another city	75.17 (12.77)		
	Tehran & my spouse other city	73.33 (12.91)		
	Other city & my spouse Tehran	67.62 (10.43)		
**Information**	TV	74.86 (13.32)	1.448	0.217
	BBC & VOA	72.63 (14.19)		
	Telegram	73.51 (12.32)		
	Ministry of Health	77.16 (13.03)		
	Else	73.88 (14.61)		
**Corona**	I catch, and my spouse did not	72.63 (14.53)	0.689	0.559
	I did not catch it, and my spouse caught	74.27 (13.42)		
	I and my spouse did not catch	74.91 (13.26)		
	I and my spouse caught	74.63 (11.86)		

*<.05

To compare the sexual relationship of Iranian people based on the constructs of the protection motivation theory, each domain's scores were standardized based on 0 to 100. Perceived response efficiency with an average of 79.12 had the highest, and perceived self-efficacy with an average of 69.92 had the lowest mean score among all constructs (Table 3).

**Table 3:** Mean and standard deviation of the sexual relationship regarding Protection Motivation (*n* = 716)

Variable	Minimum	Maximum	Mean	Std. Deviation
**Perceived vulnerability**	0	100	75.48	21.26
**Perceived severity**	0	100	72.39	23.22
**Perceived costs**	0	100	74.17	19.86
**Perceived response efficacy**	0	100	79.12	23.43
**Perceived self-efficacy**	0	100	69.92	20.27
**Protection motivation**	0	100	76.79	19.67
**Total**	7.61	100	74.55	13.32

The perceived vulnerability was significantly differenced with employment status (*p* = 0.019) and place of residence (*p* = 0.021); Post hoc analysis revealed that in people with part-time job significantly less than people with a full-time job (*p* = 0.001), unemployed (*p* = 0.013), self-employed (*p* = 0.045), housewife (*p* = 0.029) and other jobs (*p* = 0.028). Also, people who lived in the city were significantly more than people living in Tehran (*p* = 0.028), and people who lived in the city and their spouses lived in Tehran (*p* = 0.017). Perceived vulnerability also had a significant positive correlation with age; with age, perceived vulnerability also increased (*p* = 0.049). Other domains did not have a significant relationship with any of the studied units' demographic characteristics (Table 4).

**Table 4:** Separate means and SD (the numbers in parentheses are standard deviations) of 6 dimensions of Protection Motivation according to demographic characteristics and their correlation (*n* = 716)

Variables	Perceived vulnerability			Perceived severity			Perceived costs			Perceived response efficacy			Perceived self-efficacy			Protection motivation		
	**M(SD)**	T or F	P	**M(SD)**	T or F	P	**M(SD)**	T or F	P	**M(SD)**	T or F	P	**M(SD)**	T or F	P	**M(SD)**	T or F	P
**Sex**																		
Female	75.71 (21.02)	0.689	0.491	72.20 (23.25)	0.498	0.618	74.32 (19.54)	0.488	0.626	79.72 (23.09)	1.614	0.107	69.89 (20.30)	0.091	0.927	76.68 (19.53)	0.354	0.723
Male	74.18 (22.63)			73.42 (23.16)			73.30 (21.69)			75.75 (25.15)			70.09 (20.20)			77.41 (20.54)		
**Marital Status**																		
Married	75.29 (21.38)	0.755	0.47	72.10 (23.43)	0.6	0.549	74.12 (19.95)	1.993	0.137	78.94 (23.60)	0.622	0.537	69.83 (20.47)	0.992	0.371	76.66 (19.88)	0.374	0.688
Divorced & Widow	75.49 (21.91)			74.83 (21.91)			70.72 (20.29)			78.94 (22.10)			68.09 (17.96)			76.75 (19.19)		
Temporary marriage	80.72 (16.57)			76.30 (19.41)			80.98 (15.25)			84.37 (21.01)			75.26 (18.10)			80.20 (14.07)		
**Job**																		
Full-time	77.77 (19.98)	2.727	^*^0.019	71.73 (23.41)	0.725	0.605	75.28 (20.34)	0.705	0.62	80.09 (24.10)	1.964	0.082	71.64 (20.29)	2.039	0.071	76.09 (20.42)	1.893	0.093
Part-time	65.70 (24.23)			66.55 (23.85)			71.11 (15.19)			72.46 (22.93)			65.20 (16.57)			75.22 (21.01)		
Self-employment	79.61 (20.31)			72.55 (23.21)			75.81 (21.17)			86.14 (17.26)			76.63 (19.78)			78.98 (11.75)		
Else	74.23 (19.40)			72.63 (24.43)			75.08 (17.78)			75.92 (21.71)			67.56 (18.23)			77.59 (16.02)		
No job	74.92 (21.88)			73.64 (22.93)			74.09 (19.88)			82.83 (20.86)			71.00 (20.81)			82.53 (18.66)		
Housewife	74.00 (22.60)			73.87 (22.55)			72.50 (20.53)			77.69 (24.41)			67.78 (21.13)			75.17 (20.35)		
**Education**																		
Diploma	78.12 (21.56)	1.092	0.275	75.08 (21.10)			74.19 (18.91)			74.82 (23.45)			69.55 (20.82)			77.26 (17.19)		
University	75.20 (21.22)			72.09 (23.44)			74.16 (19.98)			79.59 (23.40)	1.621	0.105	69.96 (20.23)			76.74 (19.93)	0.21	0.834
**Spouse's Education**																		
Diploma	75.89 (22.06)			72.38 (24.09)			73.84 (19.68)			77.18 (23.52)			69.47 (20.58)			78.42 (18.22)		
University	75.44 (21.07)	0.227	0.821	72.41 (23.14)	0.013	0.989	74.57 (19.95)	0.393	0.694	79.75 (23.31)	1.177	0.24	70.23 (20.34)	0.398	0.691	76.28 (20.09)	1.159	0.247
**Live Location**																		
Tehran	73.41 (22.50)	3.281	^*^0.021	71.07 (24.51)	1.41	0.239	73.36 (20.76)	0.282	0.838	81.14 (22.97)	1.494	0.215	69.84 (21.75)	0.957	0.412	75.03 (21.03)	1.772	0.151
Another city	77.13 (20.32)			73.43 (22.49)			74.61 (19.72)			78.40 (23.52)			70.17 (19.53)			77.95 (18.73)		
Tehran & my spouse other city	72.79 (21.64)			72.42 (23.80)			75.73 (12.07)			75.73 (25.47)			72.05 (21.77)			70.58 (19.56)		
Another city & my spouse Tehran	63.39 (22.17)			62.50 (20.80)			72.32 (16.57)			70.53 (25.05)			61.16 (13.01)			78.57 (22.34)		
**Information Source**																		
TV	75.98 (21.38)	1.61	0.17	72.81 (23.06)	1.816	0.124	74.15 (19.96)	0.222	0.926	79.44 (23.68)	0.658	0.622	71.21 (19.34)	1.618	0.168	75.81 (19.51)	1.772	0.133
BBC & VOA	76.11 (20.61)			68.37 (25.48)			73.69 (18.64)			75.65 (25.40)			69.02 (20.19)			73.01 (19.35)		
Telegram	72.55 (22.62)			72.11 (22.90)			73.20 (20.37)			78.19 (23.25)			66.95 (20.15)			79.54 (18.17)		
Ministry of Health	79.49 (18.45)			77.21 (20.77)			75.45 (21.03)			80.33 (21.96)			72.10 (20.15)			78.76 (21.83)		
Else	73.60 (21.03)			68.84 (24.65)			75.18 (18.36)			81.43 (22.40)			67.63 (24.78)			77.48 (20.66)		
**Corona**																		
I catch and my spouse did not	74.45 (20.28)	0.533	0.66	70.60 (22.39)	0.364	0.779	71.52 (22.00)	1.963	0.118	77.23 (24.31)	0.617	0.604	66.74 (22.07)	1.964	0.118	76.13 (20.86)	1.06	0.365
I did not catch and my spouse caught	78.08 (21.77)			71.69 (24.88)			78.52 (19.60)			76.91 (24.39)			66.54 (21.18)			73.72 (21.38)		
I and my spouse did not catch	75.31 (21.54)			72.55 (23.16)			73.75 (19.59)			79.88 (23.22)			71.04 (19.96)			77.57 (18.86)		
I and my spouse caught	74.53 (19.49)			74.65 (22.75)			75.11 (18.86)			78.47 (22.76)			69.79 (18.20)			75.46 (22.16)		

*<.05

Among domains of the PMSRQ-COVID-19 that all had significant correlations with each other, just there was no significant correlation between perceived severity and perceived self-efficacy (*P* = 0.067; Table 5).

**Table 5:** Pearson correlations between the dimensions of sexual relationship regarding Protection Motivation Theory (*n* = 716)

Dimensions	8	7	6	5	4	3	2	1
**1- Perceived vulnerability**	0.074*	0.668^**^	0.169^**^	0.223^**^	0.245^**^	0.367^**^	0.425^**^	1
**2- Perceived severity**	-0.042	0.623^**^	0.155^**^	0.067	0.077^*^	0.505^**^	1	
**3- Perceived costs**	-0.072	0.661^**^	0.158^**^	0.182^**^	0.224^**^	1		
**4- Perceived response efficacy**	0.051	0.660^**^	0.340^**^	0.523^**^	1			
**5- Perceived Self-efficacy**	0.073	0.597^**^	0.225^**^	1				
**6- Protection motivation**	0.069	0.491^**^	1					
**7- Total**	0.037	1						
**8-Age**								

^*^p < 0.05; **p < 0.001

## Discussion

This study aimed to determine the sexual relationship of Iranian people based on the theory of protection motivation and its related factors during home quarantine of corona prevention. In this study, a positive and significant correlation was observed between preventive sexual behaviors in the corona period and its domains.

The present study showed that there is a statistically significant correlation between age and perceived vulnerability. The older you get, the more perceived vulnerability you have and the more protective behaviors you adopt. This may be due to higher awareness and perceived threat at older ages. Older people are more prone to underlying diseases, so preventive measures are more common among older people than younger people [21]. The present study is consistent with some studies on unsafe driving in Yazd and the study of Covid-19 disease, which show that older age is associated with more protective behaviors [14, 22].

Nevertheless, Lowe et al. among Australian university students showed that older age was associated with less protective behavior against sunlight [23]. One possible explanation could be that older people are the most affected by COVID-19. Therefore, they may perceive more vulnerability and severity in this situation**.** This is different from the results of this article, and the reason may be differences in culture and type of protective behaviors. In general, each person has a unique understanding of experiencing a particular situation that may counter their health. People's sensitivity to understand a situation or disease is very diverse [24]. If people do not feel vulnerable to a health threat, they are more likely to reject the suggested healthy behaviors. Therefore, perceived vulnerability can play a key role in people's intention to adopt and maintain healthy behavior. The perceived vulnerability had a statistically significant relationship with employment status and place of residence.

The results of our study showed that people who had a part-time employment status compared to full-time people and even the unemployed had a lower perceived sensitivity score to Covid 19. On the other hand, regarding resistance the average perceived sensitivity score of people who lived in the other cities except of Tehran was higher compared to people who lived in Tehran and ae well as people who lived in the other cities except of Tehran but their spouses were in Tehran. This statistically significant difference may be due to the fact that Tehran, as the capital of Iran, is a city with people with different demographic characteristics and a very large number of mandatory and compulsory intercity trips. One possible explanation could be Unstable conditions seem to affect perceptions of sensitivity in protective behaviors. In other words, the type of working time (part-time job) and non-permanent residence during the Corona pandemic provided a sense of confusion among participants in terms of sensitivity to protective behaviors.

In this study, the perceived vulnerability domain was most associated with preventive sexual behaviors in Corona. Consistent with the present study, Grunfeld argues that the components of Threat-Appraisal (perceived vulnerability and perceived severity) and then the components of Coping-Appraisal (perceived self-efficacy, perceived response efficiency, and perceived cost) are strong predictors of intent to engage in protective behaviors [25]. If people find themselves vulnerable to the disease, they will take more protective and preventative behaviors during the Corona period. These results are consistent with Moeini et al., Ezzati Rad et al. [26, 27]. In contrast to the present study, Sharifi Rad considers Coping-Appraisal more than Threat-Appraisal, the predictor of behavior that prevents the spread and spread of influenza A based on motivation theory [28]. Okaharu et al. also reported a low perceived vulnerability during the Covid-19 epidemic. This may be because the participants in this study thought they were not likely to be infected with Covid-19 and therefore had no motivation to follow prevention and quarantine principles [29].

According to the present study, preventive sexual behaviors in the Corona period and perceived severity are positively and significantly related. Therefore, if people are aware of this disease's consequences, they will take more protective behaviors. This is consistent with Ezzati Rad et al. and Tazval et al. [27, 30] but is different from Zare et al. [31]. Perceived severity depends on people's beliefs and the mental effects of an illness or condition and its effects on their lives. These effects can be considered as problems that may cause problems for individuals. If people believe that they are exposed to moderate or high-risk health threats, the likelihood of adopting healthy behaviors is greatly increased.

According to the present study, preventive sexual behaviors in the Corona period and perceived cost were positively and significantly related to each other, consistent with Gong et al. [20]. If people believe that they are vulnerable to a health threat, that the health threat is serious and has severe side effects, and that the cost of health advice is completely unassessed, they are more likely to adopt healthy behavior.

The present study showed that protective motivated sexual relationship had the highest mean score in the area of perceived response efficiency, which is consistent with Sharifi Rad et al.'s study on the prevention of influenza A and Leigh et al.'s study of Ebola and preventive behaviors against SARS, avian influenza, and swine flu [28, 32]. A positive correlation indicates that a person can act consistently against a health risk, which can reduce health risks, protect his or her health, and prevent the consequences of inappropriate behaviors. Therefore, in designing educational interventions, emphasis on perceived responses' self-efficacy and effectiveness is essential to reduce threats. The present study also showed that protective motivated sexual relationship had the lowest mean score in the area of perceived self-efficacy, which is not consistent with various studies that showed that self-efficacy is a very important factor in performing health behaviors [32–34], but are consistent with Morwati et al. [14]. These differences may be due to differences in the study population. Positive correlation shows that when a person seriously believes that she can reduce her health risk by adopting a healthy behavior, she behaves less inconsistently and tolerates the consequences. Perceived self-efficacy is the perception of individuals about their ability to engage in activities that enable them to control events that affect their lives [12].

In the present study, protective behaviors are shown at an optimal level that is consistent with Bashirian et al. on Covid-19 protective behaviors based on protection motivation theory among hospital staff, Ezzati Rad on predicting Covid-19 preventive behaviors based on protection motivation [27, 35].

Several studies have reported information on the positive consequences of perceived severity and perceived vulnerability of infectious diseases on individuals' goals of adhering to protective and preventive behaviors [36, 37]. Significant correlations between the constructs of conservation motivation theory and behavioral intent can predict general protective behaviors in response to the Covid-19 pandemic and similar future infectious disease threats. If people do not understand the threat and severity of the infectious disease, they can easily ignore the relevant recommendations. Overall, participants in the present study reported high levels of perceived severity, perceived vulnerability, perceived response efficiency, and perceived costs. Therefore, they reported a high level of motivation for protective and preventive behaviors in all situations. From a practical point of view, the predictive validity of protection motivation theory showed that infrastructures have a significant relationship with the intention of protective and preventive behavior. This finding suggests that future interventions should focus on general cognition by developing appropriate knowledge about the severity and vulnerability of Covid-19, improving perceived response efficiency, and self-efficacy of protective behavior through skills training.

Using online polls as the main source of data collection due to the current social distance needs may limit access to all people in the community, as it excludes people with low digital literacy or lack of smartphones. However, many studies have reported that online questionnaires have significantly higher responses than e-mail questionnaires and higher quality data in online surveys than e-mail surveys [38]. Another limitation of the study was collecting data related to behaviors through self-report, which may have led to the evaluation of biased results. Therefore, more objective examination and long-term follow-up may yield different results, and further studies are needed to measure protective behaviors. Despite the limitations, this study is the first to study the sexual relations of Iranian people based on the theory of protection motivation and its related factors during the home quarantine of corona prevention on a large number of the population. Another limitation of the study was mentioned to the sample characteristics. Most participants (85.1%) were female and (90.2%) of all participants were in university education level.

## Conclusion

Perceived vulnerability is associated with employment status and place of residence, and age. protective motivated sexual relationship is only associated with employment status. Overall, participants in the present study reported high levels of perceived severity, perceived vulnerability, perceived response efficiency, and perceived costs. Therefore, they reported a high level of motivation for protective and preventive behaviors in all situations. From a practical point of view, the predictive validity of protection motivation theory showed that infrastructures have a significant relationship with the intention of protective and preventive behavior. This finding suggests that future interventions should focus on general cognition by developing appropriate knowledge about the severity and vulnerability of Covid-19, improving perceived response efficiency, and self-efficacy of protective behavior through skills training.

## Data Availability

The datasets generated and/or analysed during the current study are not publicly available due information about the sexuality of the participants in this study contradicts the confidentiality of information but are available from the corresponding author on reasonable request.

## Abbreviation

STIs: Sexual transmitted infections
